# Understanding implicature as an inner simulation of the speaker's context retrieval

**DOI:** 10.3389/fnhum.2025.1568070

**Published:** 2025-06-05

**Authors:** Shingo Tokimoto, Naoko Tokimoto

**Affiliations:** ^1^Department of English Language Studies, Mejiro University, Shinjuku City, Tokyo, Japan; ^2^Department of Performing Arts, Shobi University, Kawagoe, Saitama, Japan

**Keywords:** context retrieval, effective connectivity, implicature, indirect utterance, inner simulation, partial directed coherence, pragmatic inference, second-order theory of mind

## Abstract

In everyday conversation, speakers often convey their intentions indirectly, requiring listeners to infer meaning beyond the literal content of the utterance. For example, the question “Do you know the way to the station?” implies a request such as “Please tell me the way to the station.” Although pragmatic inference is generally assumed to support the comprehension of such implicit intentions, the underlying neural mechanisms remain poorly understood. This study investigated the cognitive and neural processes involved in comprehending indirect utterances, using electroencephalography (EEG) recorded while participants listened to spoken dialogues. We manipulated both the contextual explicitness (explicit vs. implicit) and the temporal reference (present intention vs. past experience) of the speaker's implicit intentions. EEG analyses revealed a significant effect of contextual explicitness only in conversations involving past experiences. Specifically, in the implicit context condition relative to the explicit condition, we observed a significant positive deflection in the event-related potential and significant suppression in the θ and β frequency bands of event-related spectral perturbation. The β-band suppression was interpreted as reflecting perspective-taking by the listener. To further investigate the neural mechanisms involved, we analyzed effective connectivity among 28 regions of interest—previously identified in fMRI studies of indirect utterance comprehension—using source-localized EEG data. In the implicit context condition for past-experience conversations, we found a significant increase in information flow to the parahippocampal gyrus, suggesting a role for autobiographical memory retrieval. Multiple regression analyses showed that this connectivity was significantly associated with subscores on the Autism-Spectrum Quotient, particularly the Imagination and Communication subscales—both related to theory of mind (ToM). These findings suggest that autobiographical memory retrieval is guided by second-order ToM processes, enabling listeners to internally simulate the speaker's context retrieval. Our results challenge traditional linguistic models that conceptualize the comprehension of implicit intentions as a stepwise construction of propositional representations. Instead, they support a pragmatic inference as context search model, in which listeners actively search for a context that coherently integrates the indirect utterance with the preceding discourse.

## 1 Introduction

In everyday conversation, a speaker's intentions are often conveyed indirectly through implication. For example, in the conversation in (1), if (1-B) is speaker B's only response to speaker A's question, B is perceived as unkind, even though B is not lying. This is because A's intention is actually “please tell me the way to the station if you know it,” and B is not responding to this request.

(1)    A:   Do you know the way to the station?
         B:   Yes, I do.

An utterance in which the speaker conveys an intention beyond its literal meaning, as seen in (1-A), is generally known as an indirect utterance, and an implicit meaning such as “please tell me the way to the station” in (1-A) is known as an implicature. Implicatures are assumed to be derived from the literal meaning of an utterance and its context through pragmatic inference. However, the nature of this inference process remains poorly understood. This study experimentally investigates the psychological and neural mechanisms underlying implicature comprehension.

### 1.1 Reevaluating stepwise propositional representations

In linguistics, there is a long-standing tradition of explaining the regularity observed in linguistic phenomena by constraints imposed on combinatorial symbolic representations. These constraints are metalinguistic in nature and do not necessarily reflect real-time processing directly. Nevertheless, in psycholinguistic and neurolinguistic studies grounded in linguistic theory, real-time language processing is often conceptualized as mental computation involving symbolic representations and their sequential derivation (Dekydtspotter et al., 2024; Gibson and Warren, 2004; Nelson et al., 2017; Tamaoka, 2023). This approach is also common in the study of implicature comprehension, where propositional representations and their stepwise progression are typically assumed as part of the inferential procedure. The present study examines the validity of propositional representations and their stepwise, sequential production as an inferential mechanism for understanding indirect utterance.

In linguistic discussions of implicature comprehension, Cooperative Principle by Grice (1975) in (2) is frequently invoked as a trigger for inferencing.^1^

(2)    Cooperative principle
         Make your conversational contribution such as is required, at the stage at which it occurs, by the accepted purpose or direction of the talk exchange in which you are engaged.

The Cooperative Principle serves as a general guideline, further specified by four subprinciples, known as Grice's maxims, as presented in (3).

(3)    1.    Maxim of Quantity: Give as much information as is required and no more than is required.
         2.    Maxim of Quality: Do not say what is false or that for which you lack adequate evidence.
         3.    Maxim of Relation: Be relevant.
         4.    Maxim of Manner: Be clear, be orderly, and avoid ambiguity.

In the conversation in (4), for example, Speaker B indirectly communicates “no” as an implicature. When we actively apply the Cooperative Principle and its maxims to real-time conversational processing, the inferential steps leading from B's utterance to the intended meaning of “no” can be outlined in (5).

(4)    A:    We're having a home party this Sunday; why don't you come?
         B:    I have a graduation exam next week. (implicature: No, I won't.)

(5)    Possible outline of Speaker A's inference for understanding Speaker B's implicature under the Cooperative Principle and its maxims:
         a.    Since Speaker B has not explicitly answered “yes” or “no” to my (A's) invitation, B has not provided the information I seek. Therefore, the Maxim of Quantity is violated.
         b.    However, since B is responding to my question and actively participating in the conversation, B must still be adhering to the Cooperative Principle.
         c.    Thus, B's utterance must implicitly provide the information I am looking for.
         d.    If B has a final graduation exam next week, B will likely be too busy preparing and unable to attend the party.
         e.    Therefore, B is probably indicating that B will not come to the party. B's response to my invitation must be interpreted as “No.”

This inference is an instance of abduction, based on the assumption that Speaker B's utterance is intended as a response to the question. It can be formalized as a stepwise sequence of propositional representations. If pragmatic inference can be conceptualized as a chain of propositional representations, then it becomes possible to analyze the inferential process in terms of symbolic logic. However, this stepwise chain of symbolic representations has not yet been empirically verified. Therefore, the first research question of the present study is whether pragmatic inference in the comprehension of indirect utterances can be understood as a stepwise production of propositional representations.

In the present study, we manipulate contextual explicitness in conversations containing indirect utterances in order to vary the number of inferential steps assumed in the stepwise derivation of symbolic representations. We then examine whether neural activity corresponding to these inferential steps can be detected through the analysis of listeners' electroencephalogram (EEG) during auditory presentation of the context-manipulated conversations. For instance, in (6), Speaker C indirectly responds to a yes/no question from Speaker B, and the implicature of C's utterance is “yes.” When we actively apply the Cooperative Principle to (6), the inferential steps leading from C's utterance to the intended meaning of “yes” can be outlined in (7).

(6)    A:    Ekimae-no      hunsui-de yoji-ni
                 station-front-gen fountain-at four o'clock-at
                 machiawase-yoo.
                 let's meet
                 “Let's meet at the fountain in front of the station at 4 o'clock.”
         B:    Machiawase-no basho, wakaru?
                 meeting-gen      place do you know?
                 “Do you know the place where we're supposed to meet?”
         C:    Hunsui-wa    yuumee-dayo.
                 fountain-top famous-is
                 “The fountain is famous.” (implicature: Yes, I do.)

(7)    Possible outline for understanding Speaker C's implicature in (6):
         a.    Since Speaker C has not explicitly answered “yes” or “no” to Speaker B's question, C has not provided the information B seeks. Therefore, the Maxim of Quantity is violated.
         b.    However, since C is responding to B's question and actively participating in the conversation, C must still be adhering to the Cooperative Principle.
         c.    Thus, C's utterance must implicitly provide the information B is looking for.
         d.    C states that the fountain is famous, which presupposes that C knows the location of the fountain.
         e.    Consequently, it is inferred that C knows the meeting place, and thus C's intended answer to B's question is “yes.”

Here, when part of A's utterance in (6) is modified by replacing “hunsui” (fountain) with “hiroba” (square), resulting in A's utterance in (6)[A2] below, C's intention is still understood as “yes.” However, the context necessary for comprehending C's utterance becomes less explicit, thereby rendering C's response even more indirect. Assuming a stepwise chain of propositional representations, an additional inferential step assuming that “the fountain is in the square” must minimally be added to (7).

(6)    A2: Ekimae-no       hiroba-de yoji-ni
                station-front-gen square-at four o'clock-at
                machiawase-yoo.
                let's meet
                “Let's meet at the square in front of the station at 4 o'clock.”

Since C's utterance remains identical for both (6)[A] and (6)[A2], any difference in the number of inferential steps arising from the contrast between the explicit and implicit contexts should be reflected in the neural activity associated with the comprehension of C's utterance.

Similarly, (8) presents a conversation in which Speaker C's utterance implies “no.” The stepwise inference underlying the comprehension of C's implicature, guided by the Cooperative Principle, is outlined in (9).

(8)    A:    Samui-naa. sekiyu-stove tsukete-yo.
                 cold-is     oil stove      turn on
                 “It's cold. Turn on the oil stove.”
         B:    Tsukete-kureru?
                 turn on-give me
                 “Can you turn it on?”
         C:    Sekiyu-ga nain-da.
                 oil-nom    not exist is
                 “There is no oil.” (implicature: No, I can't.)

(9)    Possible outline for understanding Speaker C's implicature in (8):
         Steps from (a) to (c) are the same with those in (7).
         d.    C states that the there is no oil, and thus C cannot turn on the oil stove.
         e.    Consequently, it is inferred that C's intended answer to B's question is “no.”

As in the case of the conversation for meeting up in (6), when “sekiyu-stove” (oil stove) is replaced with “stove” as in (8)[A2] below, C's intention is still understood as “no.” However, since the inferential context becomes less explicit, the stepwise inference must now include at least one additional step: namely, the assumption that “the stove is an oil stove.”

(8)    A2:  Samui-naa. stove tsukete-yo.
                cold-is         stove turn on
                ‘It's cold. Turn on the stove.'

In the present study, we propose the Pragmatic Inference as Context Search model, which characterizes pragmatic inference in indirect utterance comprehension not as a derivation based on a stepwise chain of propositional symbolic representations, but rather as a process of searching for a context that appropriately integrates the indirect utterance with the preceding conversation.

For instance, the abduction illustrated for the conversation about meeting up in (7) is an inference that constructs a causal relationship, as shown in (10-a). In contrast, (10-b) describes a nearly identical situation with (10-a), but it represents a context search triggered by C's utterance.

(10)    a.    Because C knows where the fountain is, C answered that the fountain was famous.
           b.    C answered that the fountain is famous. Therefore, C knows (must know) where the fountain is.

In the context where A's utterance in (6)[A2] does not explicitly mention “the fountain,” the abductive reasoning in (10-a)—which involves stepwise production of propositional representations—would require at least one additional inferential step. Accordingly, some different neural activity is predicted due to the increased inferential demand. In contrast, from the perspective of the context search model, if the context search in (10-b) yields no meaningful difference between “the fountain in front of the station” and “the fountain located in the square in front of the station,” no change in neural activity is expected.

Likewise, the expressions corresponding to the abduction and context search for the conversation about the oil stove in (8) are presented in (11-a) and (11-b), respectively.

(11)    a.    Because C cannot turn on the oil stove, C answered that there was no oil.
          b.    C answered that there was no oil. Therefore, C is (must be) unable to turn on the oil stove.

In the context of (8)[A2], where A's utterance does not mention the “oil stove,” the stepwise abductive inference in (11-a) entails at least one additional inferential step assuming that the stove is an oil stove. Thus, a difference in neural activity is predicted due to the increased inferential demand. The precise nature of the mental and neural representation of context within the context search model remains unclear. However, in conversational situations involving the ignition of a stove on a cold day, we assume that if there is no substantial neural difference in the listener's search for a mental or neural representation of an oil stove—triggered by Speaker C's utterance “There is no oil”—between the context where (8)[A] includes “oil stove” and the context where (8)[A2] includes only “stove,” then a marked difference in neural activity between the explicit and implicit context conditions would not be expected.

### 1.2 Temporal properties of implicature

The present study also focuses on the involvement of temporal information in the comprehension of indirect utterance. Since implicature comprehension can be considered a form of mind-reading, it is naturally expected to be related to Theory of Mind (ToM). Several functional magnetic resonance imaging (fMRI) studies have examined the neural activity associated with the comprehension of indirect expressions, frequently reporting activation in the mentalizing network, including the medial prefrontal cortex (mPFC), superior temporal sulcus (STS), anterior cingulate cortex (ACC), and (right) temporoparietal junction (TPJ) (e.g., Bašnáková et al., 2014; Feng et al., 2017, 2021; Jang et al., 2013; Shibata et al., 2011; Ackeren et al., 2016). However, we should note here that several previous researches suggest that ToM may have an internal structure. For example, Komeda et al. (2016) conducted an fMRI experiment to examine the relationship between temporal and spatial processing in perspective-taking. They independently manipulated time (i.e., presence or absence of the passage of time) and location (i.e., same or different location) in experimental narratives and presented them to 21 typically developing (TD) adults and 20 adults with autism spectrum disorder (ASD) while undergoing fMRI scanning. In the different-location condition, the right TPJ exhibited greater activation in the ASD group than in the TD group, consistent with findings linking the right TPJ to spatial perspective-taking (Ferstl and Cramon, 2007). In the time-passage condition, the ACC was more activated in the TD group than in the ASD group. The ACC is involved in time perception, particularly when comparing long- and short-interval estimations (Pouthas et al., 2005). Based on these results, Komeda et al. (2016) suggested that perspective-taking was an integrated function involving both temporal and spatial information processing. Similarly, Tokimoto and Tokimoto (2018) found that neural activity associated with perspective-taking in sentence comprehension partially overlapped with that of past tense processing. These findings suggest that temporal processing plays a distinct role within the broader function of ToM. Therefore, even in implicature comprehension, where ToM is involved, variations in temporal properties are likely to influence inference processes.

We should note, however, that previous studies on indirect utterance comprehension have not adequately controlled for the temporal characteristics of experimental discourse. For example, Bašnáková et al. (2014) examined the neural activity associated with understanding direct and indirect utterances through the auditory presentation of conversations between two interlocutors to participants on the basis of fMRI data. Some experimental conversations from Bašnáková et al. (2014) are reproduced in (12). In (12), “John” and “Robert” are students attending a course in philosophy. The critical utterance by Robert, i.e., “It's hard to give a good presentation” (which is underlined in (12)), followed the different preceding contexts of (12-a) to (12-c); in particular, the critical utterance was assumed to be interpreted as a direct reply in (12-a), as an indirect informative reply in (12-b), and as an indirect face-saving reply in (12-c).

(12)    a.    Context for the direct reply
                 John: How is it to prepare a poster?
                 Robert: A nice poster is not so easy to prepare.
                 John: And how about a presentation?
                 Robert: It's hard to give a good presentation.
          b.    Context for the indirect informative reply
                 John: I think that I will rather write a paper.
                 Robert: I agree, you are a very good writer.
                 John: Will you choose a presentation?
                 Robert: It's hard to give a good presentation.
          c.    Context for the indirect face-saving reply
                 John: I'm relieved it's over!
                 Robert: Yes, the lecturer was really strict.
                 John: Did you find my presentation convincing?
                 Robert: It's hard to give a good presentation.

Bašnáková et al. (2014) identified brain regions that exhibited more activation in response to the indirect informative replies than the direct replies, regions that exhibited more activation in response to the indirect face-saving replies than the direct replies, and regions that exhibited more activation in response to the indirect face-saving replies than the indirect informative replies. The experiment conducted by Bašnáková et al. (2014) was well controlled, and their analysis was rigorous. However, the temporal property that characterizes the implicature of the utterance by Robert could differ across the three conditions. With respect to the two indirect replies in particular, the implicature in (12-b) pertains to Robert's present intention, whereas the implicature in (12-c) pertains to Robert's past experience. That is, the implicit intention of Robert in (12-b) is “I will not prepare a presentation,” whereas the corresponding intention in (12-c) is “I did not find your presentation convincing.” The temporal properties of implicature might be confounded in Bašnáková et al. (2014) and potentially in previous functional MRI experiments. Thus, the second research question of the present study is to elucidate the effect of temporal property on implicature comprehension, under the assumption that ToM may have an internal structure.

The indirect utterances in the dialogues in Japanese briefly discussed in (6) and (8) imply Speaker C's present intention (or knowledge). However, from the perspective of the pragmatic inference as context search, the temporal characteristics of the context may influence the search process. In (13), for example, the implicature of Speaker C's utterance is ‘yes,' and the stepwise inference for understanding C's implicature is outlined in (14).

(13)    A:    Hokkaidoo-ryokoo-ni ittan-dattene.
                   Hokkaidoo-trip-to      went-did you?
                   “I hear you went on a trip to Hokkaidoo.”
           B:    Ryokoo tanoshi-katta?
                   trip       enjoy did?
                   “Did you enjoy the trip?”
           C:    Hokkaidoo-wa tengoku-dane.
                   Hokkaidoo-top heaven-is
                   “Hokkaidoo is heaven.” (implicature: Yes, I did.)

(14)    Possible outline for understanding Speaker C's implicature in (13):
           Steps from (a) to (c) are the same with those in (7).
           d.    C states that Hokkaidoo is heaven, which suggests that C found Hokkaidoo comfortable.
           e.    Consequently, it is inferred that C enjoyed the trip, and thus C's intended answer to B's question is “yes.”

Replacing part of Speaker A's utterance, “Hokkaidoo” (Hokkaidoo), with “isshuukan” (one week) makes the context for inference more implicit, while Speaker C's utterance and its implicature remain the same (“Yes, I did.”) in both the explicit context in (13)[A] and the implicit context in (13)[A2].

(13)    A2: Isshuukan-ryokoo-ni ittan-dattene.
                 one week-trip-to      went-did you?
                 “I hear you went on a trip for a week.”

As in the cases of conversations about Speaker C's present intention discussed in (6) and (8), when Speaker A's utterance in (13)[A2] does not mention “Hokkaidoo,” the stepwise abduction requires at least one additional inferential step—namely, the assumption that “the one-week trip was a trip to Hokkaidoo.” Consequently, a difference in neural activity is predicted due to the increased inferential demand. It is important to note that, from the perspective of context search, the context that “the one-week trip was a trip to Hokkaido” must be retrieved from Speaker C's past experiences, which are embedded in C's knowledge.

As suggested by previous fMRI studies on indirect utterance comprehension, if ToM is involved in implicature processing, then second-order ToM may be required for context search within the speaker's prior knowledge. The second-order ToM is the ability to understand that someone else has beliefs about another person's beliefs or thoughts. It goes beyond the first-order ToM, which refers to the understanding that others can hold beliefs different from one's own. The structure of first- and second-order ToM can be linguistically represented by clause embedding, as shown in (15):

(15)    Sally thinks that Anne believes the marble is in the basket.

The production and comprehension of (15) requires recognizing not only Sally's belief, but also her belief about Anne's belief—hence, a second-order mental state. This capacity typically develops around ages 6–7 in children and is crucial for more complex forms of social reasoning, such as sarcasm, deception, and irony (Miller, 2009). In the comprehension of implicatures related to a speaker's past experience, it is necessary to search for past contextual information embedded within the speaker's knowledge. Therefore, second-order ToM can be involved.

(16) is conversation about the speaker's past experience in which C implies “no,” and the stepwise abduction for understanding C's implicature is outlined in (17).

(16)    A:    Kono-mae-no    nichiyoobi,
                   this-before-gen Sunday,
                   eigo-no shiken-dattan-desho?
                   English exam-was-is it?
                   “You had an English exam last Sunday, didn't you?”
           B:    Shiken gookaku-shita?
                   exam passing-did
                   “Did you pass the exam?”
           C:    Eigo-wa      nigate-da.
                   English-top poor at-is
                   “I'm poor at English.” (implicature: No, I didn't pass the exam.)

(17)    Possible outline for understanding Speaker C's implicature in (16):
           Steps from (a) to (c) are the same with those in (7).
           d.    C states that C is poor at English, which suggests that C did not perform well on the exam.
           e.    Consequently, it is inferred that C did not pass the exam, and thus C's intended answer to B's question is “no.”

By replacing part of Speaker A's utterance, “eigo-no shiken” (English exam), with “shiken” (exam), the context for inference becomes more implicit, while Speaker C's utterance and its implicature remain the same (“No, I didn't.”) in both the explicit context in (16)[A] and the implicit context in (16)[A2].

(16)    A2: Kono-mae-no nichiyoobi, shiken-dattan-desho?
                  this-before-gen Sunday,       exam-was-is it?
                  “You had an exam last Sunday, didn't you?”

The stepwise abduction for (16)[A2] requires at least one additional inferential step assuming that “the exam was an English exam.” Accordingly, a difference in neural activity is predicted due to the increased number of inferential steps. According to the context search model, on the other hand, the context that “the exam was an English exam” must be retrieved from Speaker C's past experience, which is embedded within Speaker C's knowledge. The second-order ToM thus can operate for the understanding C's intention, when C's utterance follows (16)[A2].

The research questions and predictions of the present study are summarized in (18).

(18)    a.    Neural activity corresponding to contextual explicitness
                 If pragmatic inference in the comprehension of indirect utterances involves the stepwise construction of propositional symbolic representations, then neural activity corresponding to the number of inferential steps should be observed when comparing the explicit and implicit context conditions, regardless of whether the implicature pertains to the speaker's present intention or past experience.
          b.    Effect of the temporal properties of implicature on contextual explicitness
                 If pragmatic inference in indirect utterance comprehension operates as a process of searching for a context that coherently integrates the indirect utterance with the preceding discourse, then no substantial difference in neural activity is predicted between the explicit and implicit context conditions in conversations about the speaker's present intention. However, in the implicit context condition of conversations about the speaker's past experience, the relevant context to be retrieved is embedded within the speaker's knowledge. Because this retrieval process is expected to involve second-order ToM, a difference in neural activity is predicted between the explicit and implicit context conditions.

To address the research questions in the present study, we conducted an experiment to examine the EEG associated with the comprehension of implicatures in conversation.

## 2 Materials and methods

### 2.1 Experimental conversations

The experimental stimuli consisted of conversations in Japanese involving three speakers. In each conversation, Speaker A introduced a topic, Speaker B posed a question related to the topic, and Speaker C responded with an indirect utterance that implied either a “yes” or “no.” Examples of the experimental conversations are presented in (19) to (22), where (19) and (20) represent conversations involving implicature about the speaker's present intention, while (21) and (22) involve implicature about the speaker's past experience. As briefly discussed in the introduction, the number of inferential steps assumed for the stepwise production of propositional expressions was manipulated through the context explicitness by modifying part of Speaker A's utterance to fit either the explicit or implicit context condition. The utterances by Speakers B and C remained identical across both conditions. The modification made to Speaker A's utterance is indicated in parentheses ({explicit context/implicit context}). In Japanese, the standard tense system consists of nonpast and past. To manipulate the temporal properties of Speaker C's implicature (i.e., present intention vs. past experience), we varied the tense of Speaker B's utterance, using nonpast for conversations about present intention and past for conversations about past experience. However, the tense of Speaker C's utterance remained nonpast across all conversations. Event markers were placed at the onset of critical words in Speaker C's utterance for EEG analysis, corresponding to the point at which Speaker C's intention could be inferred. These markers are indicated in (19) to (22) by underlining.

(19)    Conversation about present intention in which C implies “yes” ((6) in the introduction)
           A:    Ekimae-no          {hunsui/hiroba}-de
                   station-front-gen {fountain/square}-at
                   yozi-ni            machiawase-yoo.
                   four o'clock-at let's meet
                   “Let's meet at the {fountain/square} in front of the station at 4 o'clock.”
           B:    Machiawase-no-basho, wakaru?
                   meet-at-place                 do you know?
                   “Do you know the place at which we should meet?”
           C:    Hunsui-wa   yuumee-dayo.
                   fountain-top famous is
                   “The fountain is famous.” (implicature: Yes, I do.)

(20)    Conversation about present intention in which C implies “no” ((8) in the introduction)
           A:    Samui-naa. {sekiyu-stove/stove} tsukete-yo.
                   cold-is     {oil stove/stove}      turn on
                   “It's cold. Turn on the {oil stove/stove}.”
           B:    Tsukete-kureru?
                   turn on-give me
                   “Can you turn it on?”
           C:    Sekiyu-ga nain-da.
                   oil-nom not exist is
                   “There is no oil.” (implicature: No, I can't.)

(21)    Conversation about past experience in which C implies “yes” ((13) in the introduction)
           A:    {Hokkaidoo/isshuukan}-ryokoo-ni ittan-dattene.
                   {Hokkaidoo/one week}-trip-to     went-did you?
                   “I hear you went on a trip {to Hokkaidoo/for a week}.”
           B:    Ryokoo tanoshi-katta？
                   trip      enjoy did?
                   “Did you enjoy the trip?”
           C:    Hokkaidoo-wa tengoku-dane.
                   hokkaidoo-top heaven-is
                   “Hokkaidoo is heaven.” (implicature: Yes, I did.)

(22)    Conversation about past experience in which C implies “no” ((16) in the introduction)
           A:    Kono-mae-no nichiyoobi,
                   this-before-gen Sunday,
                   {eigo-no shiken/shiken}-dattan-desho?
                   {English exam/exam}-was-is it?
                   “You had an {English exam/exam} last Sunday, didn't you?”
           B:    Shiken gookaku-shita?
                   exam passing-did
                   “Did you pass the exam?”
           C:    Eigo-wa      nigate-da.
                   English-top poor at-is
                   “I'm poor at English.” (implicature: No, I didn't pass the exam.)

We conducted a preliminary experiment using a questionnaire to assess whether Speaker C's utterances were understood as intended. Additionally, because we manipulated context explicitness by modifying part of Speaker A's utterance—while keeping Speakers B and C's utterances identical—to precisely examine its effect, it was essential to ensure comparable comprehension accuracy across both context conditions for the experimental stimuli. The questionnaire included eight types of conversations, systematically varying along three factors: context explicitness (explicit vs. implicit), temporal property (present intention vs. past experience), and implicit intention (yes vs. no). A total of 54 native Japanese speakers participated, each tasked with identifying Speaker C's intended meaning as either yes or no. Conversation pairs were selected for inclusion in the experimental set if at least 75% of participants correctly identified the intended meaning in both the explicit and implicit context conditions. Additionally, we ensured that, across the full set of conversations about present intentions and past experiences, there was no significant difference in comprehension accuracy between the explicit and implicit context conditions. As a result, 31 pairs of conversations about present intention in the explicit and implicit conditions (including 17 pairs in which an answer of yes was implied and 14 pairs in which an answer of no was implied) and 29 pairs of conversations about past experience in the two context conditions (including 13 pairs in which an answer of yes was implied and 16 pairs in which an answer of no was implied) were chosen, and a total of 120 experimental conversations were counterbalanced and divided into two stimulus sets consisting of 60 conversations each. Forty conversations among three persons that did not include indirect utterances were included in the main session as fillers; thus, the main session included 100 conversations. ^2^^,^ ^3^

The stimulus conversations were synthesized using the voices of three male or three female speakers. Event markers were placed at the onset of critical words in Speaker C's utterances, marking the point at which C's intention (i.e., “yes” or “no”) could be determined, to support the EEG analysis presented below.

### 2.2 Participants

Twenty-four native Japanese speakers (10 males) aged 18–28 years (*M* = 20.13, *SD* = 2.11) participated in this study. All participants had normal or corrected-to-normal vision and no history of neurological or psychiatric disorders. Handedness was assessed using the Edinburgh Handedness Inventory (Oldfield, 1971), confirming that all participants were right-handed.

After providing informed consent, participants took part in the EEG measurement experiment, followed by the completion of the Japanese version of the Autism-Spectrum Quotient (AQ) (Wakabayashi et al., 2006). In the present study, the AQ was not used to investigate ASD but rather as an index of individual differences in sociality that may be related to pragmatic inference. The AQ consists of 50 questions divided into five subscales (10 items each), which can be further categorized into AQ-Social (Communication, Imagination, Social Skill), reflecting communication and social interaction abilities, and AQ-Attention (Attention Switching, Local Details), related to attentional control (Davis et al., 2017). In the following sections, we discuss the correlation between AQ subscale scores and neural activity as an indicator of the psychological function of the activity. A significant correlation with AQ-Social scores would suggest an association with ToM, whereas a correlation with AQ-Attention scores would indicate involvement in attentional allocation strategies. Descriptive statistics for participants' AQ responses, along with example questions, are presented in Table 1.

**Table 1:** **(A)** Descriptive statistics for participants' responses to the Japanese version of the Autism-Spectrum Quotient (AQ). **(B)** Sample questions from each of the five AQ subscales (two out of ten questions per subscale).

**(A)**	**Age**	**AQ total**	**Communication**	**Social skill**	**Imagination**	**Attention switching**	**Local details**
**14 females**							
Mean	20.14	21.36	4.00	3.43	3.36	5.43	5.14
SD	2.38	5.26	2.54	2.24	1.82	1.60	1.70
Maximum	28	31	9	8	8	8	9
Minimum	18	12	0	0	1	2	2
**10 males**							
Mean	20.10	22.40	4.10	4.20	2.50	5.80	5.80
SD	1.79	5.23	2.28	1.62	1.18	1.69	2.57
Maximum	24	30	8	7	4	8	10
Minimum	18	12	1	2	1	3	2
**(B)**							
Communication a. Other people frequently tell me that what I've said is impolite, even though I think it is polite. b. I frequently find that I don't know how to keep a conversation going.							
Social skill a. I would rather go to a library than to a party. b. I find it hard to make new friends.							
Imagination a. When I'm reading a story, I find it difficult to work out the characters' intentions. b. I like to collect information about categories of things (e.g., types of cars, birds, trains, plants).							
Attention switching a. I prefer to do things the same way over and over again. b. I frequently get so strongly absorbed in one thing that I lose sight of other things.							
Local details a. I often notice small sounds when others do not. b. I am fascinated by numbers.							

The participants were paid for their efforts. This study was approved by the Ethics Committee of Shobi University.

### 2.3 Predictions

The research questions of the present study are to ascertain whether neural activity corresponding to contextual explicitness is observed, and whether the effect of contextual explicitness varies with the temporal properties of implicature. In this study, we analyzed neural activity using scalp event-related potentials (ERPs), scalp event-related spectral perturbations (ERSPs), and effective connectivity among brain regions of interest (ROIs) derived from scalp EEG data.

#### 2.3.1 Predictions for ERPs

Previous studies on the comprehension of indirect utterances—including metaphors and irony—have frequently reported a negative ERP deflection peaking around 400 ms (the N400), generally interpreted as reflecting semantic processing (Coulson and Petten, 2002; Deckert et al., 2021; Filik et al., 2014). In addition, some studies have observed a positive ERP deflection peaking around 600 ms (the P600) in response to irony (Spotorno et al., 2013). In our study, Speaker C's utterances were indirect in both the explicit and implicit context conditions, with a greater degree of indirectness in the implicit condition. Accordingly, an N400 effect may be observed in the implicit relative to the explicit context condition. However, because our primary research goal is to examine neural activity corresponding to the degree of contextual explicitness and to explore whether this effect varies depending on the temporal properties of the implicature, we did not formulate specific predictions regarding individual ERP components. Therefore, the ERP analysis will be exploratory in nature.

#### 2.3.2 Predictions for ERSPs

Concerning the role of ToM in implicature comprehension, several EEG experiments have shown that perspective-taking is associated with β suppression. For instance, Woodruff et al. (2016) presented participants with photographs of actors displaying various emotions (happy, sad, angry, and neutral). In the “self condition,” participants indicated how the actor's emotion made them feel, whereas in the “other condition” they identified the displayed emotion. The study reported significant β enhancement at the F4, Fz, C3, C4, and Cz electrodes in the self condition, and significant β suppression at the F3 and C3 electrodes in the other condition. In contrast, Tokimoto and Tokimoto (2023) manipulated perspective-taking in sentence comprehension using two Japanese giving and receiving verbs, revealing significant β suppression at frontal and central electrodes in response to sentences involving perspective-taking (compared to those that did not) within a 200–600 ms window based on critical words. According to our Pragmatic Inference as Context Search model, the context regarding the speaker's past experience—embedded within the speaker's knowledge—is retrieved under the implicit context condition of conversations about past experience. Given that second-order ToM processes may be involved in such retrieval, we predict that β suppression—reflecting perspective-taking—will occur to a greater extent in the implicit than in the explicit context condition of conversations about past experience.

#### 2.3.3 Predictions for connectivity analysis

Recent neuroscientific research suggests that brain function involves flexible, integrated processing and that cognitive functions are supported by large-scale connectivity across brain networks (Anderson and Barbey, 2023). Accordingly, we computed source-level neural activity for ROIs identified in fMRI studies of indirect utterance comprehension by transforming scalp EEG data and analyzed effective connectivity between these ROIs. Although EEG-based connectivity analyses offer lower spatial resolution compared to fMRI, they provide key advantages: (a) the ability to analyze the temporal dynamics of connectivity over short time windows relevant to language processing (Michel and He, 2019), (b) the capacity to examine activity across different frequency bands, and (c) the ability to evaluate the directionality of information flow. While such EEG-based effective connectivity analyses in language processing remain rare, Tokimoto and Tokimoto (2023) reported interactions among the mentalizing network, mirror neuron system, and executive control network, along with their temporal dynamics across a broad frequency range (θ to γ), during perspective-taking in sentence comprehension. In line with these findings, we can expect that causal interactions among ROIs and their temporal variations will be observable in our study across frequency bands ranging from θ to γ. However, we did not formulate specific predictions regarding the connectivity, and thus our analysis of connectivity will be exploratory.

### 2.4 Procedure

**Figure 1:**
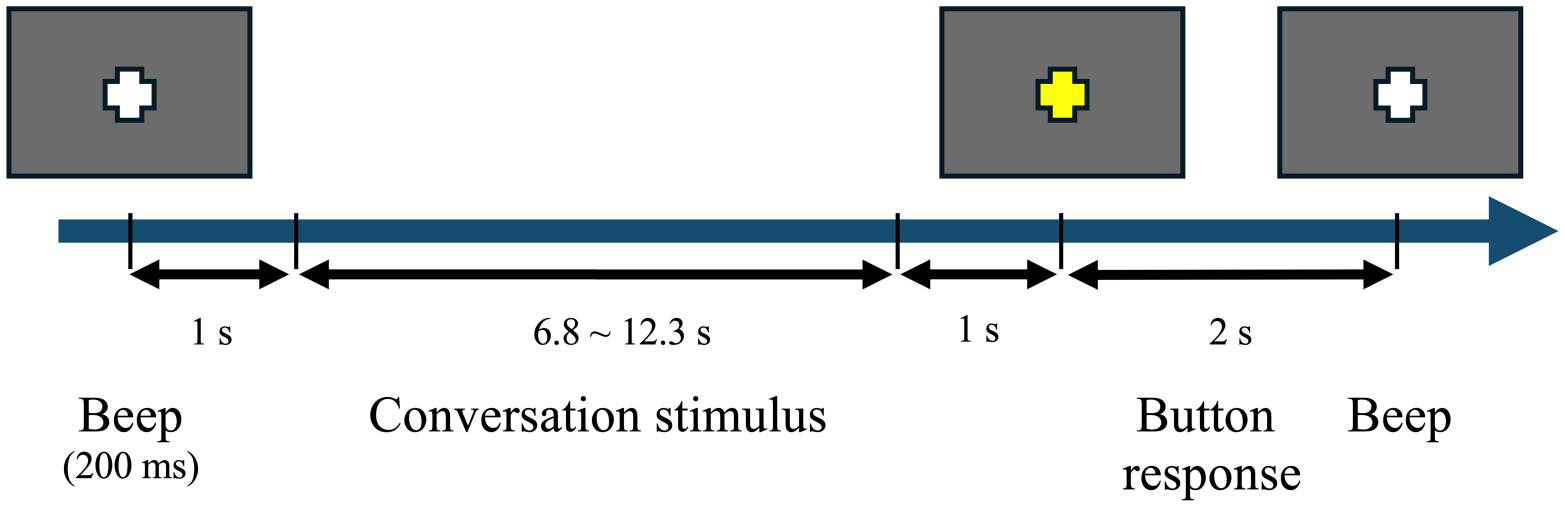
Sequence of stimuli and responses in a trial.

The participants were seated in an electrically and acoustically shielded EEG chamber 1 m in front of a 5.5-inch LCD monitor. The sound of a beep indicated the beginning of a trial, and a white fixation point was presented visually in the center of the display. A conversation stimulus was presented auditorily one second after the beep. The fixation point turned yellow one second after the end of the conversation stimulus, and the participants were asked to judge the intention of Speaker C's utterance (i.e., yes or no) by pressing one of two buttons. The lengths of the conversation stimuli ranged from 6.8 to 12.3 s; thus, each trial lasted 10.8–16.3 s. The sound stimuli were presented with ER2 Insert Earphones (Etymotic Research). Figure 1 illustrates the sequence of the experimental stimuli and the responses of the participants.

The order in which the conversation stimuli were presented was randomized for each participant. The experiment was controlled with Presentation software (Neurobehavioral Systems). The practice session consisted of four trials. The main session consisted of two blocks, and the participants were allowed to rest for 3–5 min between the blocks. The experimental sessions, including the instruction and application of the electrodes, lasted 1.5 h.

### 2.5 EEG recording

EEG signals were recorded using a 64-channel EEG amplifier (BrainAmp DC, Brain Products, Germany) and an active electrode system (actiCAP, Brain Products) configured according to the extended 10–20 system. Signals were sampled at 2.5 kHz, and a bandpass filter from 0.1 to 200 Hz was applied. The reference electrode was positioned at FCz. Vertical and horizontal electrooculograms (EOGs) were recorded simultaneously using electrodes placed below the right eye and at the outer canthus of the left eye. Electrode impedance was kept below 20 kΩ throughout the recording sessions. EEG data were continuously acquired using Brain Vision Recorder software (Brain Products). The average EEG recording duration was 24.45 min (SD = 2.69 min).

### 2.6 EEG data preprocessing

The acquired EEG data were processed offline using EEGLAB (Delorme and Makeig, 2004). The preprocessing pipeline included the following steps. (1) The data were high-pass filtered at 1 Hz with the aim of minimizing low drifts with respect to the reference at FCz. (2) Line noise was removed with the assistance of the CleanLine plugin for EEGLAB. (3) High-amplitude artifacts were removed from the EEG data via artifact subspace reconstruction (Mullen et al., 2015). (4) The data were decomposed via an adaptive mixture of independent component (IC) analyzers (AMICA) (Palmer et al., 2007). (5) The best-fitting single-equivalent current dipole was calculated for each IC with the aim of matching the scalp projection of each IC source on the basis of a standardized three-shell boundary element head model. The electrode locations were aligned according to the 10–20 system with a standard brain model (Montreal Neurological Institute). (6) The ICLabel plugin in EEGLAB was used to estimate the probabilities of the following sources for each IC: brain neural activity, EOG measurements, muscle potentials, electrocardiogram measurements, line noise, channel noise, and other sources. The classifier of ICLabel was trained on thousands of manually labeled ICs and hundreds of thousands of unlabeled ICs that were collected by the Swartz Center for Computational Neuroscience (Pion-Tonachini et al., 2017). We chose ICs for which the probability of brain neural activity was greater than 70% for the subsequent analyses. (7) ICs for which the equivalent dipole model explained <85% of the variance in the corresponding IC scalp map were excluded from the subsequent analyses. The average number of rejected ICs across the 24 participants was 48.63 (SD = 3.81). Therefore, the average number of remaining ICs was 15.38. (8) The data were segmented into time epochs extending from −2 to 3 s relative to the event markers.

## 3 Results

### 3.1 Behavioral responses

**Figure 2:**
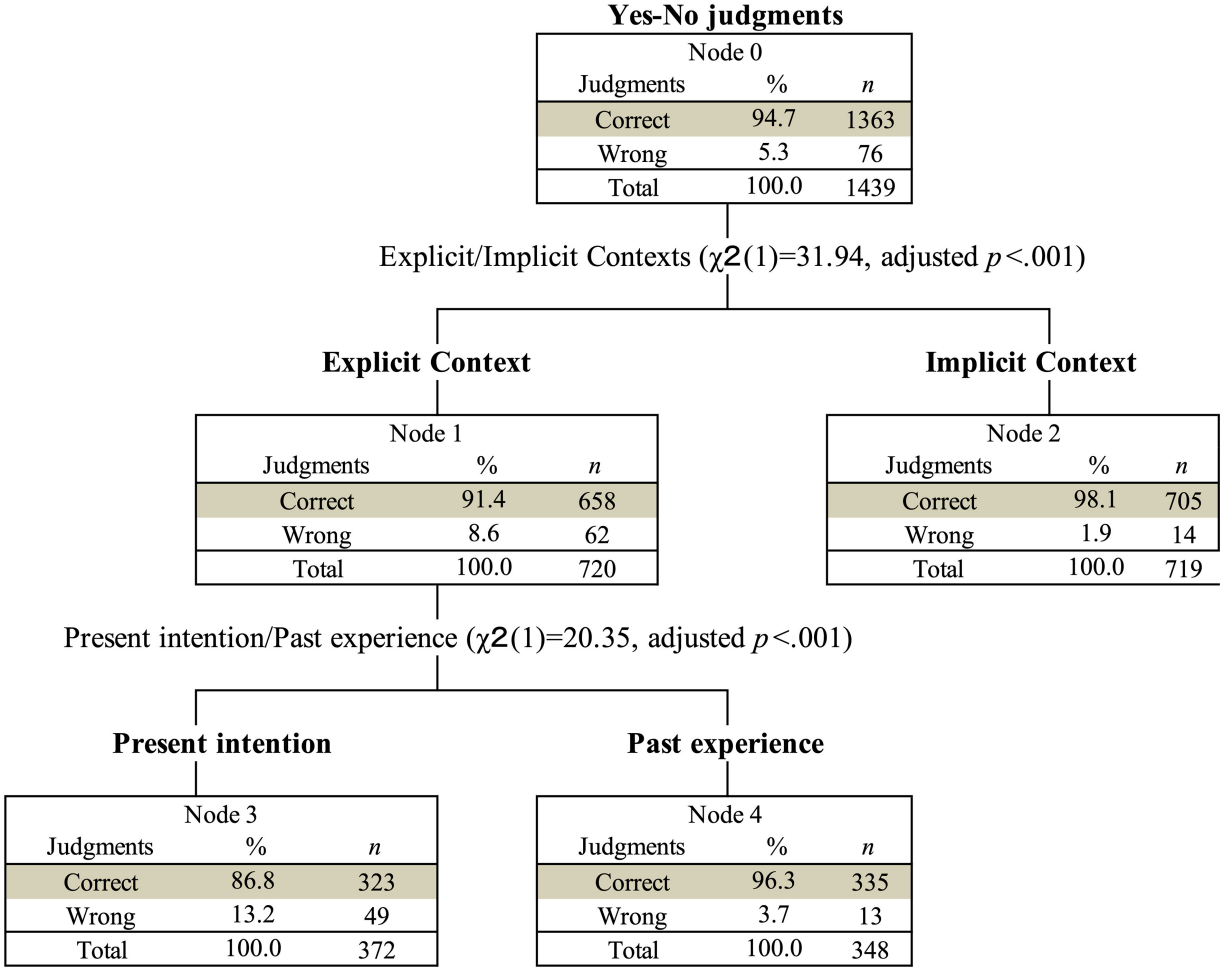
Decision tree for the yes-no judgments (correct or wrong), in which temporal property (present intention or past experience) and context explicitness (explicit or implicit context) serve as the independent variables.

Participants correctly identified the intended meaning (i.e., “yes” or “no”) of Speaker C's utterances in 94.7% of the time. Figure 2 presents a decision tree of the binary judgment outcomes (correct vs. incorrect), with temporal property (present intention vs. past experience) and context explicitness (explicit vs. implicit context) as independent variables. Overall, Speaker C's intentions were judged more accurately in conversations involving implicit context than in those involving explicit context. Specifically, under explicit context conditions, participants more accurately judged C's intentions in conversations about past experiences than in those about present intentions.

As the primary aim of the present study is to investigate the effects of contextual explicitness on implicature comprehension—and its potential modulation by the temporal properties of the implicature—we do not analyze or discuss the specific distinctions between “yes” and “no” interpretations in the subsequent sections (see text footnote 3).^4^

### 3.2 Event-related potential

To investigate potential neural correlates of the effects of temporal properties and context explicitness in conversational implicature, ERPs were analyzed using the STUDY command structure in EEGLAB. Nonparametric random permutation statistics were employed to test for significant condition effects, with multiple comparisons corrected using cluster-based permutation tests (Maris and Oostenveld, 2007). A total of 2,000 random permutations were generated and compared against the corresponding *t*-values for mean differences between conditions.

**Figure 3:**
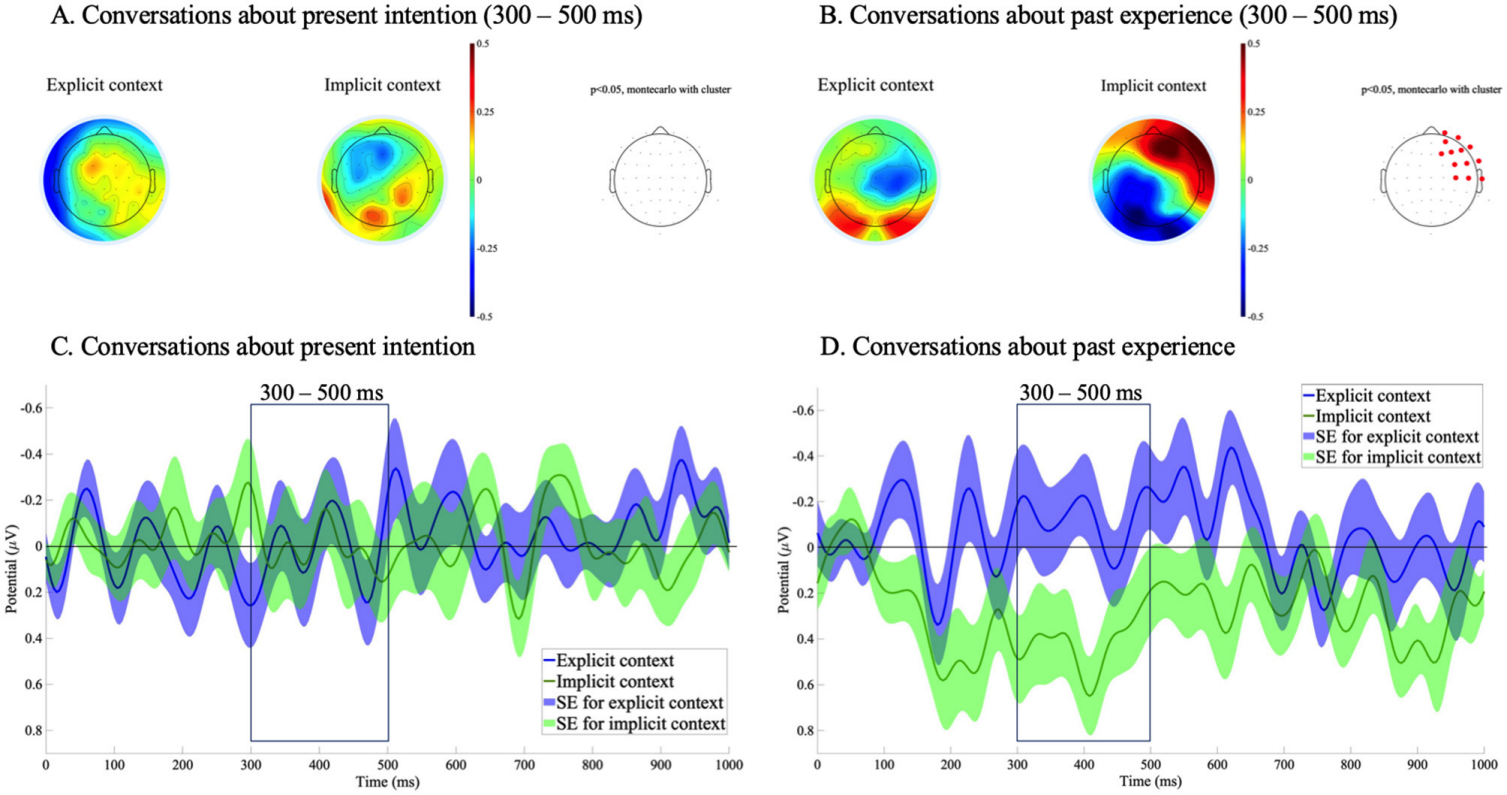
Event-related potentials (ERPs) time-locked to the onset of critical words in Speaker C's utterances, with a poststimulus baseline from 0 to 100 ms. **(A)** Mean ERP topographies from 300 to 500 ms for conversations about present intentions and **(B)** for conversations about past experiences. In **(A)** and **(B)**, the left panel presents the topography for the explicit context condition, the center panel presents the topography for the implicit context condition, and the right panel indicates electrode sites in red where significant differences were observed in the cluster-based permutation test (*p* < 0.05). **(C)** Averaged ERP waveforms from 0 to 1,000 ms at 13 sites (FP2, AF8, F8, FT8, T8, AF4, F6, FC6, C6, F2, F4, FC4, C4) with standard errors for conversations about present intentions. **(D)** Averaged ERP waveforms from 0 to 1,000 ms at the same 13 electrodes with standard errors for conversations about past experiences. In **(C)** and **(D)**, negativity is plotted upward.

ERPs were time-locked to the onset of critical words in Speaker C's utterances, with a baseline period set from 0 to 100 ms following the word onset. ERP topographies were computed in consecutive 100-ms time windows from 100 to 1,000 ms. In separate ERP analyses for conversations about present intention and past experience, a significant effect of context explicitness was observed only in the past experience condition, specifically in the 300–400 ms and 400–500 ms time windows. Accordingly, ERP comparisons for both conversation types were focused on the 300–500 ms time window, as shown in Figure 3.

A significant positive deflection was observed at right frontal electrode sites in the implicit context condition compared to the explicit context condition—but only for conversations involving past experiences. Figure 3 presents the corresponding ERP topographies and waveforms.

Since a significant effect of contextual explicitness was observed in the 300–500 ms time window for conversations about past experience, we conducted multiple regression analyses for each of the two conversation types—present intention and past experience—using data from 24 participants. The dependent variable was the mean ERP difference between the implicit and explicit context conditions (implicit – explicit) in the 300–500 ms time window, and the five AQ subscale scores were used as independent variables. The results showed a significant effect of Attention Switching in conversations about present intention (β = −0.41, *p* < 0.05), and a marginally significant effect of Communication in conversations about past experience (β = 0.35, *p* < 0.1).

### 3.3 Event-related spectral perturbation

In the same way with the ERP analysis, ERSPs were analyzed using the STUDY command structure in EEGLAB. Nonparametric random permutation statistics were employed to test the significance of condition effects, with multiple comparisons corrected using cluster-based permutation tests (Maris and Oostenveld, 2007). A total of 2,000 random permutations were generated and compared against the corresponding *t*-values of the mean condition differences. ERSPs were time-locked to the onset of critical words in Speaker C's utterances and analyzed across four frequency bands: θ (5–7 Hz), α (8–12 Hz), β (14–28 Hz), and γ (30–50 Hz), with a common baseline applied across conditions. ERSP topographies were computed in consecutive 100-ms time windows from 0 to 1,000 ms for all frequency bands.

**Figure 4:**
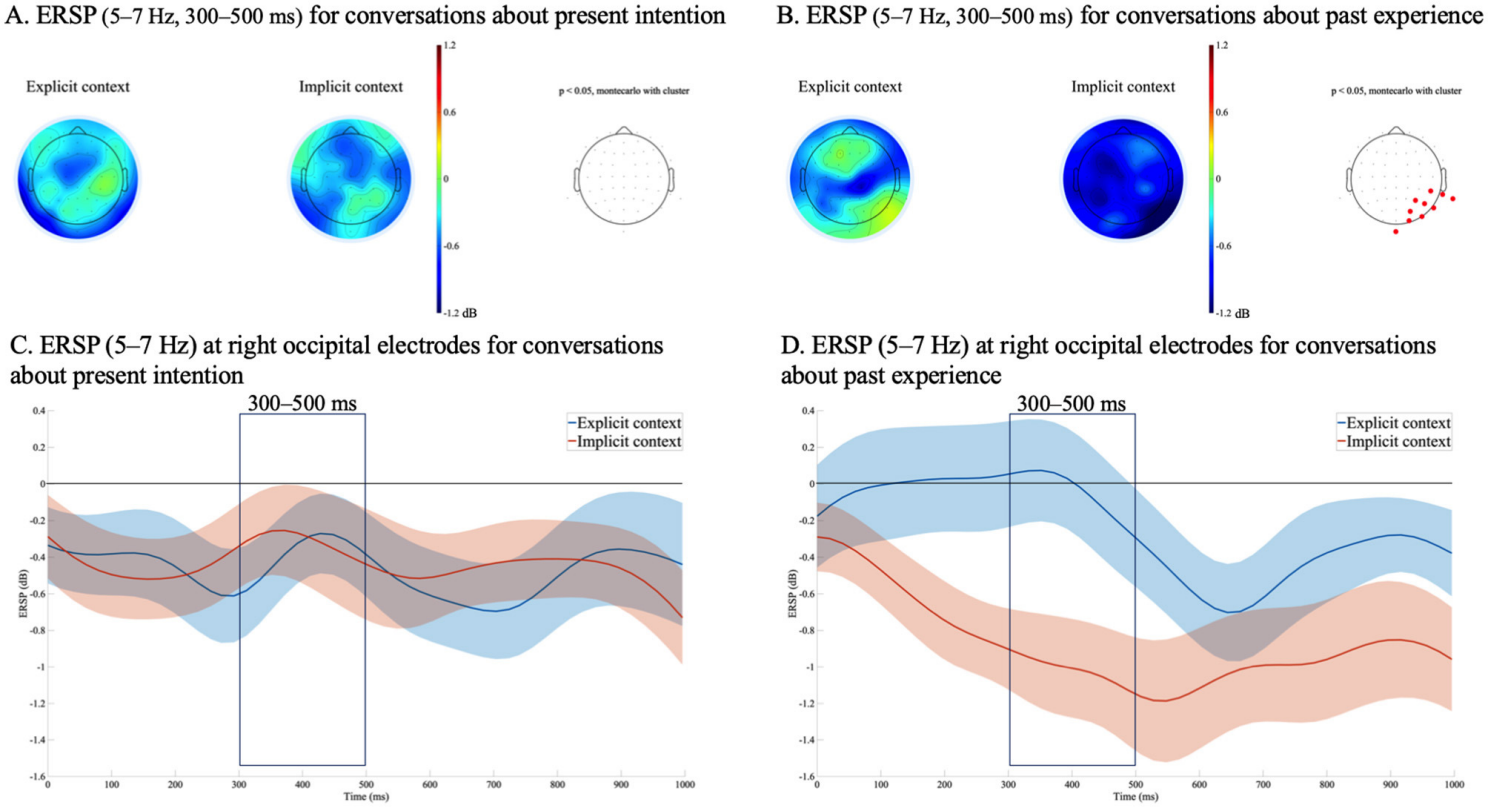
Event-related spectral perturbations (ERSPs) time-locked to the onset of the critical words in Speaker C's utterances, with a common ERSP baseline across conditions. **(A)** Mean ERSP topographies in the θ band (5–7 Hz) from 300 to 500 ms for conversations about present intentions, and **(B)** for conversations about past experiences. In **(A)** and **(B)**, the left panel presents the topography for the explicit context condition, the center panel presents the topography for the implicit context condition, and the right panel indicates electrode sites in red where significant differences were observed in the cluster-based permutation test (*p* < 0.05). **(C)** Averaged ERSP (5–7 Hz) from 0 to 1,000 ms at 10 sites (CP6, TP8, TP10, P4, P6, P8, PO4, PO8, O2, Iz) with standard errors for conversations about present intentions. **(D)** Averaged ERSP (5–7 Hz) from 0 to 1,000 ms at the same 10 electrodes with standard errors for conversations about past experiences.

**Figure 5:**
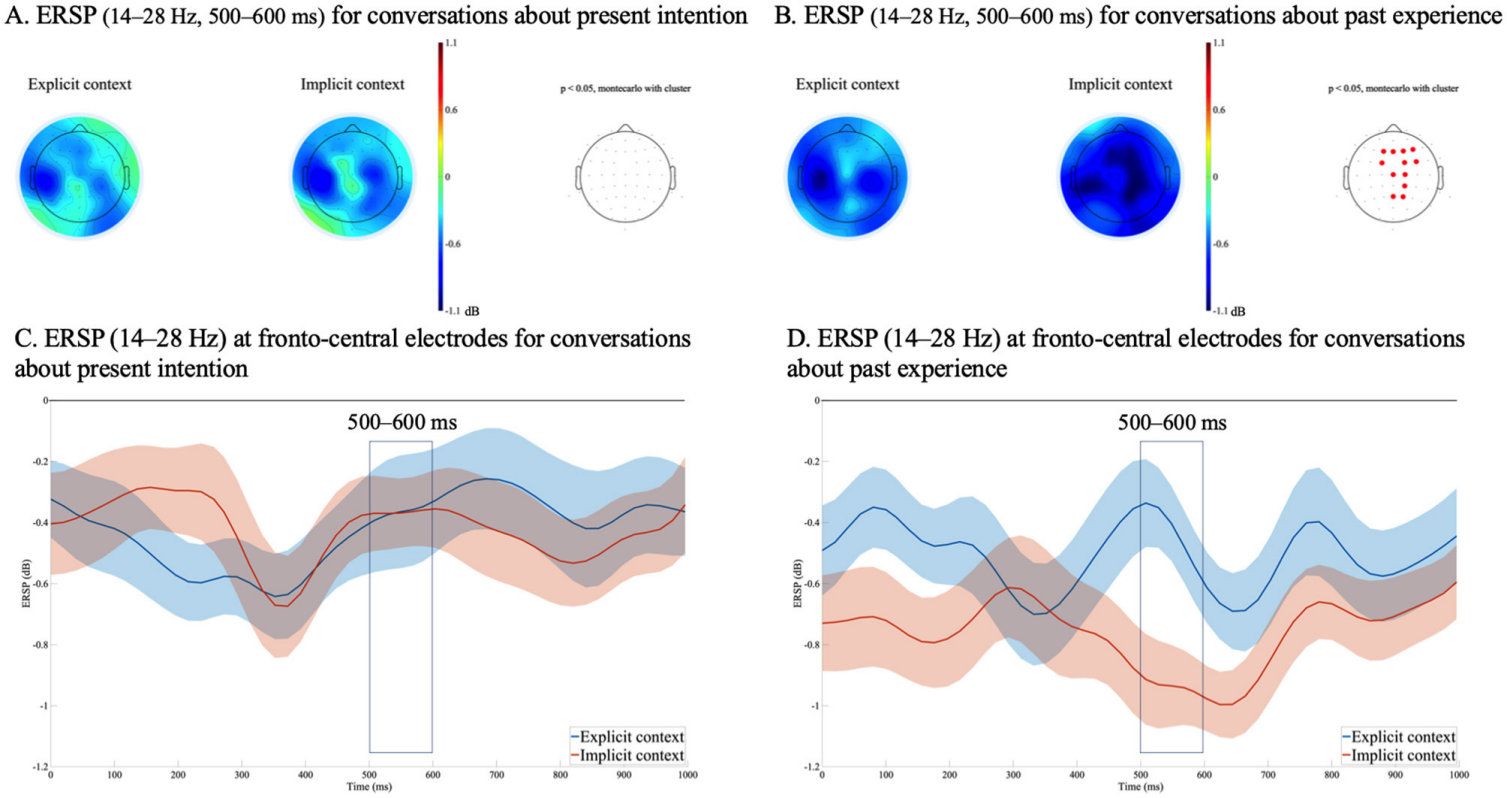
Event-related spectral perturbations (ERSPs) time-locked to the onset of the critical words in Speaker C's utterances, with a common ERSP baseline across conditions. **(A)** Mean ERSP topographies in the β band (14–28 Hz) from 500 to 600 ms for conversations about present intentions, and **(B)** for conversations about past experiences. In **(A)** and **(B)**, the left panel presents the topography for the explicit context condition, the center panel presents the topography for the implicit context condition, and the right panel indicates electrode sites in red where significant differences were observed in the cluster-based permutation test (*p* < 0.05). **(C)** Averaged ERSP (14–28 Hz) from 0 to 1,000 ms at 12 sites (F1, Fz, F3, F4, FC1, FC2, FC4, Cz, C2, CP2, Pz, P2) with standard errors for conversations about present intentions. **(D)** Averaged ERSP (14–28 Hz) from 0 to 1,000 ms at the same 12 electrodes with standard errors for conversations about past experiences.

A significant effect of context explicitness was observed in conversations about past experiences in the θ band during two time windows: 300–400 ms and 400–500 ms. Accordingly, the effect of context explicitness was examined over the 300–500 ms time window. In addition, a significant effect of context explicitness was observed in the β band, also in conversations about past experiences, during the 500–600 ms time window. Figures 4, 5 illustrate the ERSP topographies and the averaged ERSP waveforms for the θ and the β bands, respectively.

Specifically, significant θ suppression was observed in the right posterior region during the 300–500 ms time window, while significant β suppression was observed in the frontal and central regions during the 500–600 ms time window, both in the implicit context condition relative to the explicit context condition. Notably, these effects were found only in conversations concerning past experiences.

For the θ band, in the 300–500 ms time window, and for the β band, in the 500–600 ms time window, we conducted multiple regression analyses using the mean ERSP difference between the implicit and explicit context conditions (implicit – explicit) as the dependent variable, and the five AQ subscale scores as independent variables. These analyses were performed separately for conversations about present intention and those about past experience, using data from 24 participants. The results showed a significant effect of the Social Skill subscale in the θ band for conversations about past experience (β = −0.60, *p* < 0.01), while no AQ subscale showed a significant effect for conversations about present intention. In the β band, no significant effects of any AQ subscales were found for either type of conversation.^5^

### 3.4 Connectivity analyses

**Figure 6:**
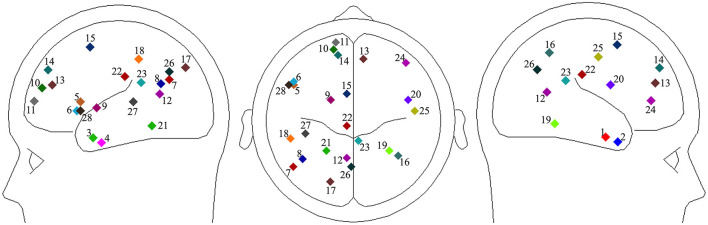
Twenty-eight regions of interest (ROIs). **(Left)** sagittal view of the left hemisphere; center: horizontal view; **(right)** sagittal view of the right hemisphere.

As the third possible manifestation of the effects of context explicitness, we analyzed effective connectivity at the source level, which may also provide insights into the neural mechanisms underlying the comprehension of implicatures. Regarding the selection of ROIs for our effective connectivity analysis, several functional MRI studies have investigated the neural processes involved in comprehending indirect expressions. In the present study, we based our ROI selection on Jang et al. (2013), who identified various brain regions whose activations were significantly correlated, either positively or negatively, with the implicitness ratings of direct and indirect answers to preceding questions. Given the possibility that reduced activation may serve a functional role in language processing, we included 25 brain regions discussed in Jang et al. (2013) in our set of ROIs. Additionally, we incorporated three ROIs from Tang et al. (2020), who examined brain regions highly relevant to time perception using linguistic expressions in Japanese, English, and Chinese as experimental stimuli. Since our study manipulated the temporal properties of conversations, these three ROIs from Tang et al. (2020) may exhibit effective connectivity with the 25 ROIs identified by Jang et al. (2013). Table 2 presents the hemisphere, structure, MNI coordinates, and Brodmann area for the 28 ROIs. R1 to R25 were drawn from Jang et al. (2013), with R1 to R15 representing regions where activation was positively correlated with implicitness rating scores, while R16 to R25 showed negative correlations. R26 to R28 were drawn from Tang et al. (2020). Figure 6 illustrates the anatomical locations of the 28 ROIs.

**Table 2:** Twenty-eight regions of interest (ROIs), including the hemisphere, structure, MNI coordinates, and Brodmann area (BA).

**Region**	**Hemisphere**	**Structure**	**MNI coordinates**			
			*x*	*y*	*z*	*B* **A**
R1	Right	Middle temporal gyrus	50	2	−24	21
R2	Right	Superior temporal gyrus	40	14	−28	38
R3	Left	Superior temporal gyrus	−48	10	−22	38
R4	Left	Middle temporal gyrus	−52	2	−26	21
R5	Left	Inferior frontal gyrus (tri)	−50	22	10	45
R6	Left	Inferior frontal gyrus (tri)	−50	26	2	47
R7	Left	Angular gyrus	−50	−66	30	39
R8	Left	Superior temporal gyrus	−42	−58	26	39
R9	Left	Putamen	−18	6	4	
R10	Left	Superior frontal gyrus	−16	60	22	10
R11	Left	Medial frontal gyrus	−14	68	10	10
R12	Left	Posterior cingulate	−4	−56	16	23
R13	Right	Superior frontal gyrus	10	50	24	9
R14	Left	Superior frontal gyrus	−12	54	38	9
R15	Left	Superior frontal gyrus	−4	12	58	6
R16	Right	Inferior parietal lobule	40	−54	50	40
R17	Left	Precuneus	−18	−82	40	19
R18	Left	Inferior parietal lobule	−52	−36	48	40
R19	Right	Fusiform gyrus	32	−48	−12	37
R20	Right	Inferior frontal gyrus	48	6	22	44
R21	Left	Parahippocampal gyrus	−22	−48	−12	37
R22	Left	Cingulate gyrus	−4	−22	32	23
R23	Right	Cingulate gyrus	6	−38	26	31
R24	Right	Middle frontal gyrus	46	46	8	46
R25	Right	Precentral gyrus	54	−6	46	4
R26		Precuneus	0	−66	36	7
R27	Left	Heschl	−40	−30	10	41
R28	Left	Broca	−54	22	2	45

To analyze the effective connectivity at the source level, we transformed the EEG data we obtained from the electrode space into the source space with BESA Research (version 7.1, BESA GmbH) in light of the montages of the 28 ROIs. The source waveforms were calculated in line with the principle of a generalized montage, in which weights were assigned to all sixty-two channels on the scalp. The transformation was based on the scalp topographies that resulted from focal brain activities (e.g., from dipole and volume conductor modeling) as well as on the principles of linear algebra, resulting in an efficient spatial filter (Michel and He, 2019; Scherg et al., 2002, 2019). To measure the directed information flow among the 28 ROIs, we calculated the partial directed coherence (PDC) in the frequency domain with BESA Connectivity (version 2.0, BESA GmbH). The PDC is a multivariate directional connectivity measure that reflects the direct interrelations among signals (Baccalá and Sameshima, 2001). The magnitude of the PDC is defined as


(1) $$\begin{array}{l} |{\text{PDC}}_{ij}\left(f\right)|=\frac{\left|\Lambda_{ij}\left(f\right)\right|}{\sqrt{\sum_{k=1}^{N}|\Lambda_{kj}\left(f\right){|}^{2}}} \end{array}$$

where Λ_*ij*_(*f*) is an element of Λ(*f*) = H^−1^(*f*).

PDC_*ij*_(*f*) describes the directional flow of information between the *j*th and *i*th signals (*j* → *i*). The PDC is normalized to take values ranging between 0 and 1. The transmission ratio from signal *j* to signal *i* and the total outflow from signal *j* (i.e., the sum along the columns of Λ(*f*)) are thus obtained (BESA GmbH, 2023). The PDC is assumed to be more efficient in computational terms as well as more robust than the directed transfer function because the former does not involve any matrix inversion (Cao et al., 2021; He et al., 2014). Our EEG data at the source level were first transformed into the time-frequency domain via the complex demodulation method. Complex demodulation is a technique that can be used to describe the amplitude and phase of a given frequency component of a time series as functions of time, thus providing a uniform frequency resolution across the bandwidth under analysis (Hao et al., 1992). In the present study, the PDC was computed in the frequency domain via a nonparametric spectral factorization approach in the θ (5–7 Hz), α (8–12 Hz), β (14–28 Hz), and γ (30–50 Hz) bands.

#### 3.4.1 Effective connectivity among 28 ROIs

Since we observed a significant positive ERP deflection in the 300–500 ms time window, as well as significant θ and β suppressions in the 300–500 ms and 500–600 ms time windows, respectively, in the implicit context condition relative to the explicit context condition for conversations about past experiences, we calculated the partial directed coherence (PDC) for consecutive 100-ms time windows from 300 to 600 ms. This analysis aimed to examine potential changes in effective connectivity within this time window.

The mean PDCs for each of the two context conditions (explicit and implicit) were computed separately for each participant and for each type of conversation (i.e., conversations about present intentions and those about past experiences). PDC values were calculated for the θ, α, β, and γ frequency bands in consecutive 100-ms time windows from 300 to 600 ms. Across the 378 ROI pairs within the 28 ROIs, PDC values were computed in both directions, resulting in 756 PDC values per time window and frequency band. These values were compared between the explicit and implicit context conditions using paired-sample *t*-tests, with multiple comparisons corrected via nonparametric cluster-based permutation testing (*N* = 1,000 permutations) (Maris and Oostenveld, 2007).

**Figure 7:**
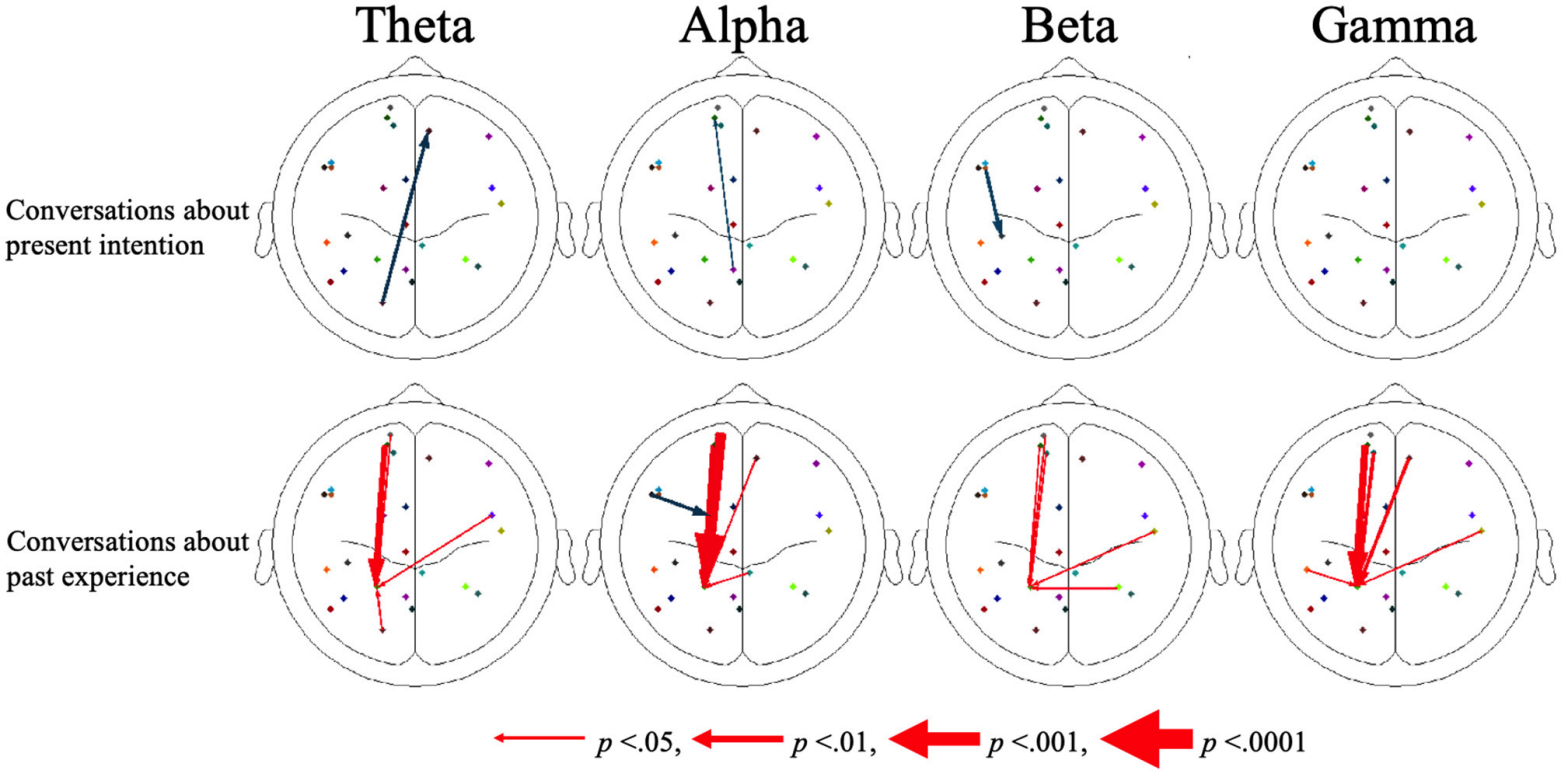
Pairs of regions of interest (ROIs) showing significant differences in mean PDC values between the implicit and explicit context conditions (implicit minus explicit) in the 400–500 ms time window across the four frequency bands. The top row depicts results from conversations about present intentions, while the bottom row depicts those from conversations about past experiences. Arrows indicate the direction of information flow; arrow width reflects the level of statistical significance. Red arrows represent a significant increase in PDC in the implicit relative to the explicit context condition, whereas blue arrows represent a significant decrease.

Pairs of ROIs where the difference between the mean PDC values in the implicit and explicit context conditions (i.e., mean PDC in the implicit condition minus mean PDC in the explicit condition) was significant across the three time windows and four frequency bands are presented in Table 3 for conversations about present intentions and in Table 4 for conversations about past experiences. Figure 7 schematically illustrates ROI pairs where the difference in PDC values between the implicit and explicit context conditions was significant in the 400–500 ms time window across the four frequency bands. As shown in Tables 3, 4 and Figure 7, when comparing increases and decreases in information flow related to contextual explicitness between conversations about present intentions and those about past experiences, we observed that increased information flow in the implicit context condition for past-experience conversations was predominantly concentrated in the left parahippocampal gyrus (R21) from multiple ROIs.

**Table 3:** Pairs of ROIs and their corresponding brain structures showing significant differences in mean PDC values between the implicit and explicit context conditions (implicit minus explicit) for conversations about present intentions, across the three time windows and four frequency bands.

**300–400 ms**	**400–500 ms**	**500–600 ms**
θ **(5–7 Hz)**		
	• R13 ←^**^ R17 r_SFG; l_precuneus	• R10 ←^*^ R25 l_SFG; r_PCG
		• R26 ⇐^*^ R22 precuneus; l_cingulate gyrus
α **(8–12 Hz)**		
	• R10 ←^*^ R12 l_SFG; l_PC	• R10 ←^**^ R16 l_SFG; r_IPL
β **(14–28 Hz)**		
• R6 ←^**^ R3 l_IFG; l_STG	• R27 ⇐^**^ R5 l_Heschl; l_IFG	• R10 ←^*^ R16 l_Heschl; l_IFG
•− ←^*^ R25 −; r_PCG		• R20 ←^*^ R15 r_IFG; l_SFG
γ **(30–50 Hz)**		
		• R26 ⇐^*^ R21 precuneus; l_PHG

For example, “R13 ←^**^ R17” indicates that the information flow from R17 (left precuneus, l_precuneus) to R13 (right superior frontal gyrus, r_SFG) was significantly lower in the implicit than in the explicit context condition at *p* < 0.01. Conversely, “R26 ⇐^*^ R22” indicates that the information flow from R22 (left cingulate gyrus, l_cingulate gyrus) to R26 (precuneus) was significantly higher in the implicit than in the explicit condition at *p* < 0.05. A “−” indicates the same ROI and structure as previously listed.

^*^p < 0.05, ^**^p < 0.001.

r_, right, l_, left; AG, angular gyrus; IFG, inferior frontal gyrus; IPL, inferior parietal lobule; PC, posterior cingulate; PCG, precentral gyrus; PHG, parahippocampal gyrus; SFG, superior frontal gyrus; STG, superior temporal gyrus.

**Table 4:** Pairs of ROIs and their corresponding brain structures showing significant differences in mean PDC values between the implicit and explicit context conditions (implicit minus explicit) for conversations about past experiences, across the three time windows and four frequency bands.

**300–400 ms**	**400–500 ms**	**500–600 ms**
θ **(5–7 Hz)**		
	• R21 ⇐^***^ R10 l_PHG; l_SFG	
	•− ⇐^*^ R17 −; l_precuneus	
	•− ⇐^*^ R11 −; l_MFG	
	•− ⇐^*^ R20 −; r_IFG	
α **(8–12 Hz)**		
	• R21 ⇐^****^ R11 l_PHG; l_MFG	
	• R9 ←^**^ R28 l_putamen; l_Broca	
	• R21 ⇐^**^ R10 l_PHG; l_SFG	
	•− ⇐^*^ R13 −; r_SFG	
	•− ⇐^*^ R14 −; l_SFG	
	•− ⇐^*^ R23 −; r_cingulate gyrus	
β **(14–28 Hz)**		
• R20 ⇐^*^ R4 r_IFG; l_MTG	• R21 ⇐^*^ R19 l_PHG; r_fusiform gyrus	
	•− ⇐^*^ R14 −; l_SFG	
	•− ⇐^*^ R25 −; r_PCG	
	•− ⇐^*^ R10 −; l_SFG	
	•− ⇐^*^ R11 −; l_MFG	
γ **(30–50 Hz)**		
	• R21 ⇐^***^ R10 l_PHG; l_SFG	
	•− ⇐^**^ R13 −; r_SFG	
	•− ⇐^**^ R14 −; l_SFG	
	•− ⇐^*^ R18 −; l_IPL	
	•− ⇐^*^ R25 −; r_PCG	

Notations are consistent with those used in Table 3.

^*^p < 0.05, ^**^p < 0.001, ^***^p < 0.0001, ^****^p < 0.00001.

r_, right; l_, left; IFG, inferior frontal gyrus; IPL, inferior parietal lobule; MFG, medial frontal gyrus; MTG, middle temporal gyrus; PCG, precentral gyrus; PHG, parahippocampal gyrus; SFG, superior frontal gyrus.

Specifically, in the 400–500 ms time window, information flow to the left parahippocampal gyrus increased from the left superior frontal gyrus (in the θ, α, β, and γ bands), the left medial frontal gyrus (in the θ, α, and β bands), the right superior frontal gyrus (in the α and γ bands), the left superior frontal gyrus (in the α, β, and γ bands), the left precuneus (in the θ band), the left inferior parietal lobule (in the γ band), the right fusiform gyrus (in the β band), the right inferior frontal gyrus (in the θ band), the right cingulate gyrus (in the α band), and the right precentral gyrus (in the β and γ bands). In contrast, for conversations about present intentions, no significant increase in information flow to the parahippocampal gyrus was observed in the implicit context condition relative to the explicit context condition.

Regarding the three ROIs previously identified as relevant to temporal perception (Tang et al., 2020), we observed significant increases in information flow in the implicit context condition relative to the explicit context condition for conversations about present intentions. Specifically, in the 400–500 ms time window, information flow increased from the left inferior frontal gyrus (R5) to the left Heschl's gyrus (R27) in the β band. Additionally, in the 500–600 ms time window, information flow increased from the left cingulate gyrus (R22) to the precuneus (R26) in the θ band and from the left parahippocampal gyrus (R21) to the precuneus (R26) in the γ band. Conversely, for conversations about past experiences, a significant decrease in information flow was observed from the left Broca's area (R28) to the left putamen (R9) in the α band in the implicit context condition relative to the explicit context condition in the 400–500 ms time window.

#### 3.4.2 Individual differences in sociality with respect to effective connectivity

To examine the effects of individual differences in sociality on effective connectivity, we conducted multiple regression analyses on pairs of ROIs that showed significant increases or decreases in PDC, as indicated in Tables 3, 4. For each participant, the dependent variable was the difference in PDC values between the implicit and explicit context conditions (implicit minus explicit), and the independent variables were the participant's scores on the five subscales of the AQ. The subscales identified as significant predictors in the regression analyses are presented in Table 5 for conversations about present intention and in Table 6 for conversations about past experience.

**Table 5:** Pairs of ROIs for which the subscales of the Autism-Spectrum Quotient (AQ) showed significant effects in multiple regression analyses.

**300–400 ms**	**400–500 ms**	**500–600 ms**
θ **(5–7 Hz)**		
	• r_SFG (R13) ←^**^ l_precuneus (R17) Attention switching (−0.395^*^)	
α **(8–12 Hz)**		
	• l_SFG (R10) ←^*^ l_PC (R12) Communication (−0.384^*^) Attention switching (0.379^*^)	• l_SFG (R10) ←^**^ r_IPL (R16) Social skill (0.484^*^)
β **(14–28 Hz)**		
• l_IFG (R6) ←^*^ r_PCG (R25) Local details (−0.397^*^) Attention switching (0.519^**^)		• r_IFG (R20) ←^*^ l_SFG (R15) Local details (0.411^*^)

In these analyses, the dependent variables were the differences in PDC values between the implicit and explicit context conditions (implicit minus explicit) for conversations about present intentions, across 28 participants. The independent variables were participants' scores on the five AQ subscales. For each ROI pair, the subscales identified as significant predictors are listed below, along with their standardized partial regression coefficients and significance levels (in parentheses). No significant effects of AQ subscales were observed for any ROI pairs in the γ band.

^*^p < 0.05, ^**^p < 0.001.

r_, right; l_, left; IFG, inferior frontal gyrus; IPL, inferior parietal lobule; PC, posterior cingulate; PCG, precentral gyrus; SFG, superior frontal gyrus.

**Table 6:** Pairs of ROIs for which the subscales of the Autism-Spectrum Quotient (AQ) showed significant effects in multiple regression analyses.

**400–500 ms**
θ (5–7 Hz)
• l_PHG (R21) ⇐^*^ r_IFG (R20) Imagination (0.401^*^)
α (8–12 Hz)
• l_PHG (R21) ⇐^****^ l_MFG (R11) Imagination (0.414^*^)
•− ⇐^*^ r_SFG (R13) Communication (-0.540^**^) Social skill (0.417^*^)
•− ⇐^*^ l_SFG (R14) Communication (-0.401^*^)
β (14–28 Hz)
• l_PHG (R21) ⇐^*^ r_fusiform gyrus (R19) Imagination (0.526^*^)
•− ⇐^*^ l_SFG (R14) Communication (-0.480^*^)
•− ⇐^*^ r_PCG (R25) Imagination (0.540^**^)
•− ⇐^*^ l_MFG (R11) Imagination (0.499^*^)
γ (30–50 Hz)
• l_PHG (R21) ⇐^**^ r_SFG (R13) Communication (-0.413^*^)
•− ⇐^**^ l_SFG (R14) Imagination (0.432^*^)
•− ⇐^*^ r_PCG (R25) Imagination (0.652^**^)

In these analyses, the dependent variables were the differences in PDC values between the implicit and explicit context conditions (implicit minus explicit) for conversations about past experiences, based on data from 24 participants. The independent variables were participants' scores on the five AQ subscales. For each ROI pair, the subscales identified as significant predictors are listed below, along with their standardized partial regression coefficients and significance levels (in parentheses). No significant effects of AQ subscales were observed for any ROI pairs in the 300–400 ms and 500–600 ms time windows.

^*^p < 0.05, ^**^p < 0.001, ^****^p < 0.00001. r_, right; l_, left; IFG, inferior frontal gyrus; MFG, medial frontal gyrus; PC, posterior cingulate; PCG, precentral gyrus; PHG, parahippocampal gyrus; SFG, superior frontal gyrus.

As shown in Table 5, for conversations about present intention in which the effect of contextual explicitness was significant, the AQ subscales most frequently associated with differences in effective connectivity across ROI pairs were Attention Switching and Local Details—both closely related to attentional allocation (Davis et al., 2017). Specifically, Attention Switching was significantly correlated with: connectivity from the right precentral gyrus to the left inferior frontal gyrus (β band; 300–400 ms), connectivity from the left precuneus to the right superior frontal gyrus (θ band; 400–500 ms), and connectivity from the left posterior cingulate to the left superior frontal gyrus (γ band; 400–500 ms). The Local Details subscale was significantly correlated with: connectivity from the right precentral gyrus to the left inferior frontal gyrus (β band; 300–400 ms), and connectivity from the left superior frontal gyrus to the right inferior frontal gyrus (β band; 500–600 ms).

In contrast, as shown in Table 6, for conversations about past experience, the AQ subscales most frequently associated with connectivity differences were Imagination and Communication—both of which are strongly linked to ToM (Davis et al., 2017). In these conversations, all significantly correlated connections converged on the left parahippocampal gyrus as the destination during the 400–500 ms time window. The sources of the connections significantly correlated with the Imagination subscale included: the left medial frontal gyrus (α and β bands), the left superior frontal gyrus (γ band), the right fusiform gyrus (β band), the right inferior frontal gyrus (θ band), and the right precentral gyrus (β and γ bands). The sources of the connections significantly correlated with the Communication subscale were: the right superior frontal gyrus (α and γ bands), and the left superior frontal gyrus (α and β bands).

## 4 Discussion

The research questions of the present study were to determine whether neural activity corresponding to contextual explicitness can be observed, and whether the effect of contextual explicitness varies with the temporal properties of implicature. In our experiment, the effect of contextual explicitness was observed only in conversations about past experiences—specifically, in the implicit context condition compared to the explicit context condition. This finding aligns with the predictions of the Context Search model (Hypothesis (18)[b]), but not with those of the stepwise symbolic model (Hypothesis (18)[a]). In this condition, a significant positive ERP deflection was observed in the right frontal region, along with significant ERSP suppression in the θ band in the right posterior region and in the β bands in the fronto-central region. In contrast, no significant effects of contextual explicitness were observed in conversations about present intentions. The finding that the effect of contextual explicitness emerged only in conversations about past experiences supports the prediction made by the Pragmatic Inference as Context Search model. Based on this, we argue that pragmatic inference in implicature comprehension is not achieved through a stepwise construction of propositional representations, but rather through the context search mechanism proposed in the present study. To the best of our knowledge, this is the first study to find significant differences in neural activity in the analyses of conversations that involve past and those that involve present.

The present study suggests that the effect of contextual explicitness observed in the implicit context condition of conversations about past experiences reflects the retrieval of the speaker's past experiences embedded within the speaker's background knowledge. We obtained evidence supporting the involvement of ToM in this context search process. In particular, the significant β suppression observed in the implicit compared to the explicit context condition for conversations about past experiences suggests that ToM—specifically, perspective-taking—was engaged during context retrieval. Furthermore, multiple regression analyses of the mean ERP difference between the implicit and explicit context conditions for conversations about past experiences revealed a marginally significant effect of the Communication subscale of the AQ. Similarly, regression analyses of the mean ERSP difference in the θ band showed a significant effect of the Social Skill subscale. In addition, multiple regression analyses of differences in PDC between the implicit and explicit context conditions for conversations about past experience revealed significant correlations with the Imagination and Communication subscales across multiple ROI pairs. The Communication, Social Skill, and Imagination subscales are grouped under AQ-Social (Davis et al., 2017), which reflects communicative and social interaction abilities deeply relevant to ToM. Taken together, the observed associations between AQ-Social scores and neural activity further support the involvement of ToM processes in the comprehension of implicatures in the implicit context condition of conversations about past experience.

An important finding concerning context search in the implicit context condition of conversations about past experiences is that many of the information flows showing significant increases in PDC for the implicit relative to the explicit context condition were directed toward the parahippocampal gyrus. Given that the parahippocampal gyrus is a key region involved in autobiographical memory retrieval (Bayley et al., 2005; Nadel et al., 2007; Viard et al., 2007), these increased flows can be interpreted as reflecting the retrieval of autobiographical memory by the comprehender. The θ suppression observed in the same condition provides converging evidence. Numerous studies have suggested a relationship between θ oscillations and memory processes, with some reporting that decreases in θ power are associated with the encoding and retrieval of episodic memory (Herweg et al., 2020). Accordingly, the θ suppression observed in the present study may likewise be interpreted as reflecting autobiographical memory retrieval during the implicit context condition of past-experience conversations. Moreover, these increases in information flow were significantly correlated with the Communication and Imagination subscales of the AQ. The significant correlations between these flow increases to the parahippocampal gyrus and the two AQ subscales suggest that the comprehender's autobiographical memory retrieval was driven by ToM processes. Based on these findings, we propose that the comprehender's self-retrieval of autobiographical memory—during the interpretation of the speaker's implicit intentions regarding past experiences—constitutes an internal simulation of the speaker's context retrieval at the time of the utterance. This simulation process is understood to be a function of ToM (Iacoboni, 2009; Oberman and Ramachandran, 2007; Schmidt et al., 2021; Schulte-Rther et al., 2007).

In contrast, in the multiple regression analyses of the mean ERP difference between the implicit and explicit context conditions for conversations about present intentions, we found a significant effect of the Attention Switching subscale. Additionally, in the multiple regression analyses of significant PDC differences for conversations about present intention, the Attention Switching and Local Details subscales were significantly correlated with the majority of significant decreases in information flow. Attention Switching and Local Details are categorized under AQ-Attention, which is closely associated with attentional allocation (Davis et al., 2017). Furthermore, in the PDC analysis for conversations about present intentions, no significant increase in information flow to the parahippocampal gyrus was observed in the implicit context condition relative to the explicit condition. These findings suggest that the process of implicature comprehension in the implicit context condition for conversations about present intentions is qualitatively distinct from that for conversations about past experiences, where ToM processes play a central role.

## 5 Limitations and future prospects

### 5.1 Mechanism for context search

The present study argues that context search constitutes the core of pragmatic inference. However, the mechanisms underlying context retrieval remain poorly understood. According to Relevance Theory, potential contexts are evaluated in the order of their “accessibility” during utterance processing (Sperber and Wilson, 2002). This notion of “accessibility” can be interpreted as reflecting associative processes, yet its precise cognitive and neural underpinnings remain unclear. While the theory of spreading activation is widely accepted in studies of lexical priming (McNamara, 1992), it remains uncertain how associative processes operate within the broader semantic space relevant to indirect utterance comprehension—particularly with regard to not only individual word meanings, but also the propositional content of utterances, world knowledge, and autobiographical memory. Moreover, some empirical findings challenge the foundational assumptions of spreading activation models (Berkum. et al., 1999).

Tokimoto (2022) identified several unresolved issues in the interpretation of indirect utterances. Two of these are particularly relevant: the retrieval of context and the convergence of inference.

Regarding context retrieval, the context necessary for understanding an implicature is not known in advance of the utterance. In principle, the number of potential contexts is infinite. These may include prior discourse, aspects of the physical environment (e.g., date, time, season, location, presence of others), and the beliefs or assumptions held by the interlocutors. Retrieving the context relevant to a given implicature could therefore involve substantial computational demands. Nevertheless, implicature comprehension typically occurs within approximately one second, suggesting that certain constraints or mechanisms must facilitate the remarkably rapid identification of relevant context.

As for the convergence of inference, miscommunication can arise for various reasons. In some cases, the comprehender fails to arrive at the intention implied by the speaker; in others, the comprehender may infer unintended implicatures that go beyond what the speaker intended. Given the theoretically infinite space of retrievable context, the number of potential implicatures could increase without bound. Consequently, misunderstandings of indirect utterances could occur frequently. However, in practice, implicatures are generally interpreted in accordance with the speaker's intended meaning. These observations suggest the existence of cognitive mechanisms that constrain the range of plausible interpretations. It is therefore reasonable to assume that the human mind and brain are equipped with mechanisms that limit or terminate context search to support appropriate and efficient communication.

Although the present study emphasizes the role of autobiographical memory in implicature comprehension, a comprehensive understanding of the mechanisms underlying context retrieval remains a key objective for future research.

### 5.2 Appropriateness of indirect utterance

The overall accuracy rate for comprehending the intended meaning (yes or no) in the experimental conversations of this study was high at 94.7%, indicating that participants generally understood the conversations as intended. As one of the reviewers correctly pointed out, the accuracy rate for comprehending the speaker's intention was higher in the implicit context condition (98.1%) than in the explicit context condition (91.4%). Consequently, the positive ERP deflection, θ suppression, and β suppression observed in the implicit context condition of conversations about past experiences cannot be attributed solely to increased processing load under the implicit context condition.

The exact reason for the higher intention comprehension accuracy in the implicit context condition remains unclear. However, one possible explanation relates to the appropriateness of indirect utterances in conversational contexts. Specifically, Speaker C's utterance in the implicit context condition was more indirect than in the explicit context condition. While indirect utterances are generally less efficient in terms of information transmission, it is possible that, depending on the conversational topic, an indirect utterance may be perceived as more “natural” than a direct one.

The appropriateness of indirect utterances in conversation has been examined within various theoretical frameworks. According to Politeness Theory proposed by Brown and Levinson (1978), strategies that save “face” in social interaction are of central importance, and indirect utterances are considered an effective means of maintaining harmonious interpersonal relationships. In contrast, Pinker et al. (2008), adopting a game-theoretic perspective, argue that indirect utterances provide strategic advantages to the speaker, yielding a higher “payoff” than direct utterances. Furthermore, cultural differences may influence how the appropriateness of indirect utterances is perceived, adding an additional layer of complexity to this issue.

This question is theoretically linked to the broader inquiry of why indirect utterances—despite their reduced efficiency in information transfer—are frequently used in verbal communication. Addressing this question is beyond the scope of the present study, and further empirical investigation is required to elucidate these mechanisms.

### 5.3 Switching of tense in conversation

In the present study, the temporal property of implicature - specifically, whether it reflected a speaker's present intention or past experience - was manipulated through the tense of Speaker B's question, which was either in the non-past or past tense, while Speaker C's utterances were consistently in the non-past tense. In conversations about past experience, therefore, the tense of utterance could be shifted from the present to the past at Speaker B's utterance.

From the perspective of discourse comprehension within a situation model framework, the situation model at the end of Speaker B's utterance in the conversation of past experience could encompass multiple temporal dimensions, whereas in the conversation of present intention, it would likely involve a single temporal dimension. A situation model incorporating two temporal dimensions might influence the processing of Speaker C's utterance differently than a model with a single temporal dimension.

The present study argues that autobiographical memory is recruited in the interpretation of implicatures arising in conversations about a speaker's past experiences. However, it is also possible that the interpretation of Speaker C's intention in the current study should be understood within the broader situation model of the entire conversation (Zwaan and Radvansky, 1998; Zwaan et al., 2000). Although the structure of the situation model preceding an indirect utterance may influence its interpretation, the internal structure of such situation models—particularly those involving implicatures—and their impact on subsequent language processing lie beyond the scope of the present study. These issues warrant further investigation in future research.

### 5.4 Constraints on fully factorial manipulation of temporal and contextual variables

To rigorously evaluate the role of temporal characteristics in the inferential processing underlying implicature comprehension, it would be ideal to compare conversations concerning present intentions and those involving past experiences under both explicit and implicit context conditions. Achieving this, however, would require constructing four distinct conversational contexts for each indirect utterance, representing all combinations of temporal property and context explicitness. Such a design would enable a full factorial manipulation of implicature type and allow for the analysis of main effects and potential interactions between these two factors. In practice, however, it is highly challenging to construct four naturalistic conversational contexts in which a single indirect utterance can plausibly be interpreted in four distinct ways, while also maintaining sufficient conversational diversity for experimental validity. Consequently, the present study prioritized the detection of context explicitness effects by manipulating this factor independently within conversations about present intentions and those about past experiences. Our analysis and discussion of temporal effects, therefore, focus primarily on the patterns observed within each of these two temporal conditions.

## 6 Conclusion

Linguistic studies have often posited a stepwise construction of symbolic representations to explain real-time language processing. However, our findings do not support this account in the context of indirect utterance comprehension. Although this view predicts a neural effect of contextual explicitness—reflecting variation in the number of inferential steps—for both conversations about present intentions and those about past experiences, we observed a significant neural effect only in the latter. Instead, the present study supports a pragmatic inference as context search model, in which comprehenders actively seek a context that allows an indirect utterance to be coherently integrated into the preceding discourse. Notably, when the implicature concerns the speaker's past experiences, comprehenders appear to recruit autobiographical memory as part of a second-order ToM process. This suggests that context retrieval in implicature comprehension may partially reflect an internal simulation of the speaker's own context retrieval at the time of the utterance.

## Data Availability

The raw data supporting the conclusions of this article will be made available by the authors, without undue reservation.

## Footnotes

^1^As one of the reviewers correctly notes, Grice did not necessarily assume that the sequence of propositional representations discussed below corresponds to real-time processing procedures. In what follows, we demonstrate the potential applicability of Grice's theory to real-time processing as one example of inference based on a sequential chain of symbolic representations.

^2^As one of the reviewers correctly pointed out, the number of conversations about present intentions and past experiences was not equal. Initially, we prepared 90 conversation pairs as candidates for the experimental set. However, based on the results of a preliminary experiment, only 60 pairs met the inclusion criteria, resulting in an unequal distribution: 31 pairs for present intentions and 29 pairs for past experiences. These 60 conversation pairs (120 conversations in total) were counterbalanced for the temporal characteristics of implicature, context explicitness, and Speaker C's intended meaning (“yes” or “no”) to create two sets of 60 conversations. Consequently, each participant was exposed to 31 conversations about present intentions and 29 conversations about past experiences during the experiment. In this study, we prioritized controlling for comprehension accuracy to precisely examine the effect of context explicitness, rather than equalizing the number of conversations across conditions.

^3^Shibata et al. (2011) conducted one of the earliest fMRI studies examining neural activity in the context of comprehending indirect expressions in Japanese. Their findings provided strong evidence supporting the claim that the mentalizing network is deeply involved in understanding implicature. However, all target stimuli in their study's indirect replies carried negative connotations, whereas those in direct (literal) replies had positive connotations. As Shibata et al. (2011) correctly noted, when participants were asked to judge a target sentence as positive or negative, their evaluation of the emotional state in the given context could influence the observed activation patterns. To control for the possibility that evaluating a speaker's emotional state might affect corresponding brain activity, we included an equal number of indirect utterances implying “yes” and those implying “no” in the experimental conversations.

^4^As discussed in the following sections, the present study attempts to interpret the psychological function of the observed neural activity differences between the explicit and implicit context conditions by analyzing their correlations with AQ subscales. However, we did not analyze the correlation between judgment accuracy and AQ subscales, as the overall accuracy for judging Speaker C's intended answer (Yes or No) was very high (94.7%), with many participants achieving 100% accuracy in both conditions, resulting in a highly skewed distribution.

^5^As one of the reviewers correctly pointed out, in the explicit context condition, part of Speaker A's utterance was repeated by Speaker C, potentially priming the processing of A's utterance. However, in the behavioral task assessing Speaker C's intention (yes or no), comprehension accuracy was higher in the implicit context condition than in the explicit context condition. Therefore, it is difficult to conclude that processing load was lower in the explicit context condition than in the implicit context condition. While the precise impact of word-level priming on sentence and discourse processing remains unclear, it is likely that the temporal characteristics of inference, rather than priming, were the primary factor driving the effect of context explicitness observed in conversations about past experiences. For a discussion on the role of priming in indirect utterance comprehension, see also the subsection of Mechanism for Context Search.
